# Treatment patterns for patients with *BRCA1/2*-positive metastatic castration-resistant prostate cancer

**DOI:** 10.1093/oncolo/oyae183

**Published:** 2024-07-31

**Authors:** Mehmet A Bilen, Ibrahim Khilfeh, Carmine Rossi, Laura Morrison, Lilian Diaz, Annalise Hilts, Patrick Lefebvre, Dominic Pilon, Daniel J George

**Affiliations:** Emory University School of Medicine, Atlanta, GA, United States; Janssen Scientific Affairs, LLC, Horsham, PA, United States; Analysis Group, Inc., Montréal, QC H3B 0M7, Canada; Analysis Group, Inc., Montréal, QC H3B 0M7, Canada; Analysis Group, Inc., Montréal, QC H3B 0M7, Canada; Analysis Group, Inc., Montréal, QC H3B 0M7, Canada; Analysis Group, Inc., Montréal, QC H3B 0M7, Canada; Analysis Group, Inc., Montréal, QC H3B 0M7, Canada; Duke University Cancer Center, Durham, NC 27710, United States

**Keywords:** androgen deprivation therapy, advanced therapy, *BRCA*-positive, metastatic castration-resistant prostate cancer, treatment patterns, treatment outcomes

## Abstract

**Background:**

Patients with *BRCA*-positive metastatic castration-resistant prostate cancer (mCRPC) have an aggressive disease course. This study aimed to describe real-world treatment patterns among patients with *BRCA*-positive mCRPC.

**Materials and methods:**

De-identified electronic health record data from the Flatiron Health-Foundation Medicine Inc. Metastatic Prostate Cancer Clinico-Genomic Database (January 01, 2011 to June 30, 2022) were used to select patients with *BRCA*-positive mCRPC initiating first-line (1L) therapy with an oncologist-defined advanced line of therapy (LOT) or androgen deprivation therapy (ADT) monotherapy. Treatment sequences and reasons for censoring were described in 1L, and among patients who initiated a second-line (2L) therapy.

**Results:**

A total of 98 treated patients with *BRCA*-positive mCRPC were identified. The top 3 treatment regimens in 1L, overall, were ADT monotherapy (19%), enzalutamide (14%), and olaparib (13%). The main reason for censoring patients with ADT monotherapy was death (52.6%). Among 79 patients treated with an advanced LOT in 1L, 43.0% (*n* = 34) did not initiate a 2L therapy, of which, 29.4% died. In patients who initiated a 2L (*n* = 45), the most common 1L to 2L treatment sequence was olaparib to docetaxel (11.1%). The most prescribed 2L therapies were docetaxel (22.2%), olaparib (20.0%), abiraterone acetate (13.3%), and enzalutamide (11.1%). From 1L initiation, the median time-to-next-treatment was 6.2 months.

**Conclusion:**

Among patients with *BRCA*-positive mCRPC, ADT monotherapy, enzalutamide, and olaparib were most commonly used. Prognosis of *BRCA*-positive patients was poor, with most patients failing initial therapy resulting in a switch to a new therapy or death. These findings highlight the need for earlier and more effective treatments for patients with *BRCA*-positive mCRPC.

## Teaser

Patients with BRCA-positive metastatic castration-resistant prostate cancer have an aggressive disease course. This article describes real-world treatment patterns among these patients.

**Implications for practice**

This study reports on treatment patterns and sequences for adult men with *BRCA*-positive metastatic castration-resistant prostate cancer (mCRPC) using linked genomic and electronic health data from oncology practices in the US. This study is one of the first to report on real-world outcomes among patients with *BRCA*-positive mCRPC, an indication for which new targeted therapies are now emerging. The study results suggest an unmet need for more effective treatment options for patients with newly diagnosed *BRCA*-positive mCRPC in clinical practice.

## Introduction

Prostate cancer (PC) is the most common non-cutaneous cancer affecting men in the US, with over 299 000 new cases estimated for 2024.^1,2^ Although the overall 5-year relative survival rate for PC is high, estimated at 97.1% between 2013 and 2019,^3^ 5-year relative survival in patients with metastatic prostate cancer (mPC) is only approximately 30%-34%.^3,4^ mPC is the second most common cause of cancer-related mortality among men in the US, accounting for 5.7% of all male cancer deaths in the US between 2013 and 2019.^1,3^ Analysis of recent trends indicates that the incidence of mPC increased substantially in the US in the years from 2010 to 2018 compared with the period from 2004 to 2010, when rates remained stable; the increase coincided with the 2012 US Preventive Services Task Force recommendations against routine prostate-specific antigen (PSA) screening across races and age groups.^5^ It is likely that the trend of increasing incidence will continue from 2018 onward, as the recommendations are still in place.

Androgen deprivation therapy (ADT) is the mainstay of treatment for patients with newly diagnosed mPC, using either bilateral orchiectomy or luteinizing hormone-releasing hormone agonists and antagonists.^6,7^ Although initial ADT can effectively reduce testosterone to castrate levels (<50 ng/dL) and prevent tumor growth and division of malignant cells, referred to as the castration-sensitive PC (CSPC) phase, eventually most patients will develop resistance to ADT and progress to castration-resistant PC (CRPC) despite castrate testosterone levels.^8,9^ The eventual progression from PC to the most aggressive form, metastatic CRPC (mCRPC) can occur through different disease stages and the prognosis is poor; mCRPC remains incurable and median survival estimates in the US range from approximately 11 to 30 months.^10-12^

The heterogeneity in outcomes for mCRPC is notable and is thought to be driven in large part by variability in the underlying molecular genetic features that affect disease trajectory.^13^ The genetic heterogeneity underlying mCRPC has become more clear in recent years with advances in next-generation DNA sequencing, allowing for targeting of molecular genetic defects with specific treatments using a personalized therapeutic approach.^14,15^ The most important category of genomic (ie, somatic and germline) mutations in PC are alterations in DNA damage repair genes, including those involved in DNA homologous recombination repair (HRR), found in 5% of less advanced localized disease and in up to 30% of mPC.^14-17^ The most common HRR alterations in mCRPC are found in *BRCA2*, and a recent systematic literature review reported germline and somatic *BRCA2* alterations occurring in 3.3%-6.0% and 5.0%-15.1% of patients, respectively.^18^ Although the prognosis for patients with alterations in *BRCA2* (and the related *BRCA1*) is worse than for patients without the alterations,^19,20^*BRCA* alterations predict response to treatment with poly ADP-ribose polymerase (PARP) inhibitors, a new class of targeted treatment for tumors, including mCRPC.^21-24^

Given the utility of genetic testing in screening, risk assessment, and management for patients with mCRPC, guidelines and recommendations from national cancer and professional organizations, including the National Comprehensive Cancer Network, the European Society for Medical Oncology, and the American Urological Association now include recommendations for somatic and germline testing for HRR alterations, including *BR*CA.^15,25,26^ Despite this information, there is limited real-world evidence on medication use and treatment patterns among patients with mCRPC who have a *BRCA1 or BRCA2* alteration (hereafter “*BRCA-*positive”) in the US. To address this evidence gap, the objectives of this study were to describe real-world treatment patterns and outcomes among patients with *BRCA*-positive mCRPC.

## Materials and methods

### Data source

This study used electronic health record (EHR)-derived data from the nationwide (US-based) Flatiron Health-Foundation Medicine, Inc. (FMI) Metastatic PC Clinico-Genomic Database (hereafter “CGDB”; 1/1/2011-6/30/2022) to identify *BRCA-*positive patients who initiated an oncologist-defined advanced line of therapy (LOT) on or after mCRPC diagnosis, or who used ADT monotherapy at the time of mCRPC diagnosis. The EHR-derived database is a longitudinal database comprising de-identified patient-level structured and unstructured data from ~280 US cancer clinics (~800 sites of care) and curated via technology-enabled abstraction.^27^ The database included a sample of patients with chart-confirmed metastatic PC (International Classification of Diseases, Ninth/Tenth Revision, Clinical Modification [ICD-9-CM: 185.x or ICD-10-CM: C61.x]) with at least 2 documented clinical visits in the Flatiron Health network, on different days, occurring on or after 1/1/2011. Retrospective longitudinal clinical data from the EHR-derived database were linked to genomic data derived from FMI comprehensive genomic profiling (CGP) tests (ie, FoundationOne, FoundationOne CDx, FoundationOne Liquid, FoundationOne Liquid CDx, FoundationOne Heme) in the CGDB by de-identified, deterministic matching.^28,29^ Genomic alterations were identified via CGP of  >300 cancer-related genes on FMI’s NGS test and include tumor specimen features (eg, mutation burden, purity), alteration-level details (eg, reference and alternate alleles, allele frequency) obtained through tissue and/or liquid biopsy, and therapeutic recommendations reported to the physician at the time of testing.^30–32^ All data were de-identified and Health Insurance Portability and Accountability Act compliant. The research was conducted according to the principles of the Declaration of Helsinki. Flatiron Health, Inc. and FMI did not participate in data analyses.

### Study design

A retrospective longitudinal cohort study design was used. First-line (1L) therapy was defined as the start date of the first oncologist-defined advanced LOT (ie, an LOT containing androgen receptor signaling inhibitors, chemotherapies, estrogens, immunotherapies, PARP inhibitors, or radiopharmaceuticals, with or without ADT) initiated on or after the date of mCRPC diagnosis, or an ADT treatment episode of any length overlapping with the date of mCRPC diagnosis, for patients without an advanced oncologist-defined LOT at any time (ie, patients using ADT monotherapy). For patients with an advanced LOT, the index date was defined as the start of 1L therapy. For patients using ADT monotherapy at mCRPC diagnosis, the index date was defined as the date of mCRPC diagnosis. Baseline patient characteristics were evaluated in the 12 months preceding the index date. Treatment patterns and sequences were assessed during the observation period which spanned from the index date until the end of clinical activity or data availability (6/30/2022). Patients were classified as *BRCA*-positive based on test results obtained prior to or on the index date, except for patients using ADT monotherapy, where patients were classified as *BRCA*-positive based on testing results observed at any time (ie, prior to or after the index date).

### Inclusion criteria

Patients were included in the study if they had a chart-confirmed diagnosis of metastatic PC; had confirmed CRPC based on a Flatiron Health algorithm incorporating (1) physician-reported CRPC in medical chart, (2) observed rising PSA values while on hormone therapy, or (3) physician documented rising PSA on hormone therapy plus a change in treatment; and were ≥18 years of age at mCRPC diagnosis (ie, latter of the date of chart confirmed metastasis or CRPC). Patients with an advanced LOT were required to have initiated ≥1 LOT for mCRPC on or after 1/1/2019. An oncologist-defined, rule-based LOT consisted of treatment for mCRPC with advanced PC medications, excluding ADT. The advanced LOTs were renumbered such that 1L was defined as the line started on or after mCRPC diagnosis. LOTs were defined as beginning on the date of treatment initiation that followed or was on the same date as mCRPC diagnosis, treatments initiated within 28 days were considered part of that LOT. The LOT end date was defined as the earliest of the day before the start date of the next LOT (if observed), the day before the last record in the database observed across all component tables, or the end of data availability. For the ADT monotherapy cohort, patients were required to be using ADT on the mCRPC date, while not initiating an advanced LOT on or after mCRPC diagnosis. Included patients were also required to have ≥12 months of clinical activity prior to the index date, and ≥1 *BRCA*-positive test prior to or on the index date (patients with an advanced LOT) or observed at any time (ADT monotherapy patients).

### Exclusion criteria

Patients with an advanced LOT were excluded from the study if they received a clinical trial medication as part of 1L therapy for mCRPC. Patients with ADT monotherapy in 1L were excluded if they were initiated on an advanced LOT after the date of mCRPC diagnosis. Patients excluded from the ADT monotherapy subgroup on this basis would be considered for the subgroup of patients with an advanced LOT in 1L, if the LOT was initiated on or after 1/1/2019.

### Study outcomes

Treatment sequences from 1L through subsequent lines (up to 3L; if observed) were assessed. Reasons for censoring (ie, death, loss to follow up, and end of data availability) between LOTs were also assessed. Time-to-next treatment (TTNT) was defined as the time from 1L initiation (index date) to the start of second-line (2L) therapy, including the use of clinical trial medication, among patients who initiated an advanced LOT.

### Statistical analysis

Patient demographic characteristics were described, overall, and for the advanced LOT and ADT monotherapy subgroups using means, standard deviations, and medians for continuous variables, and frequencies and proportions for binary variables. Kaplan-Meier analysis was used to assess the median TTNT and the proportion of patients initiating a subsequent line of therapy up to 12 months post-index, excluding ADT monotherapy patients. TTNT analysis was censored at the earliest of end of clinical activity (including death), or end of data availability. All analyses were descriptive (ie, no formal statistical testing was performed).

## Results

### Baseline characteristics

**Figure 1.**
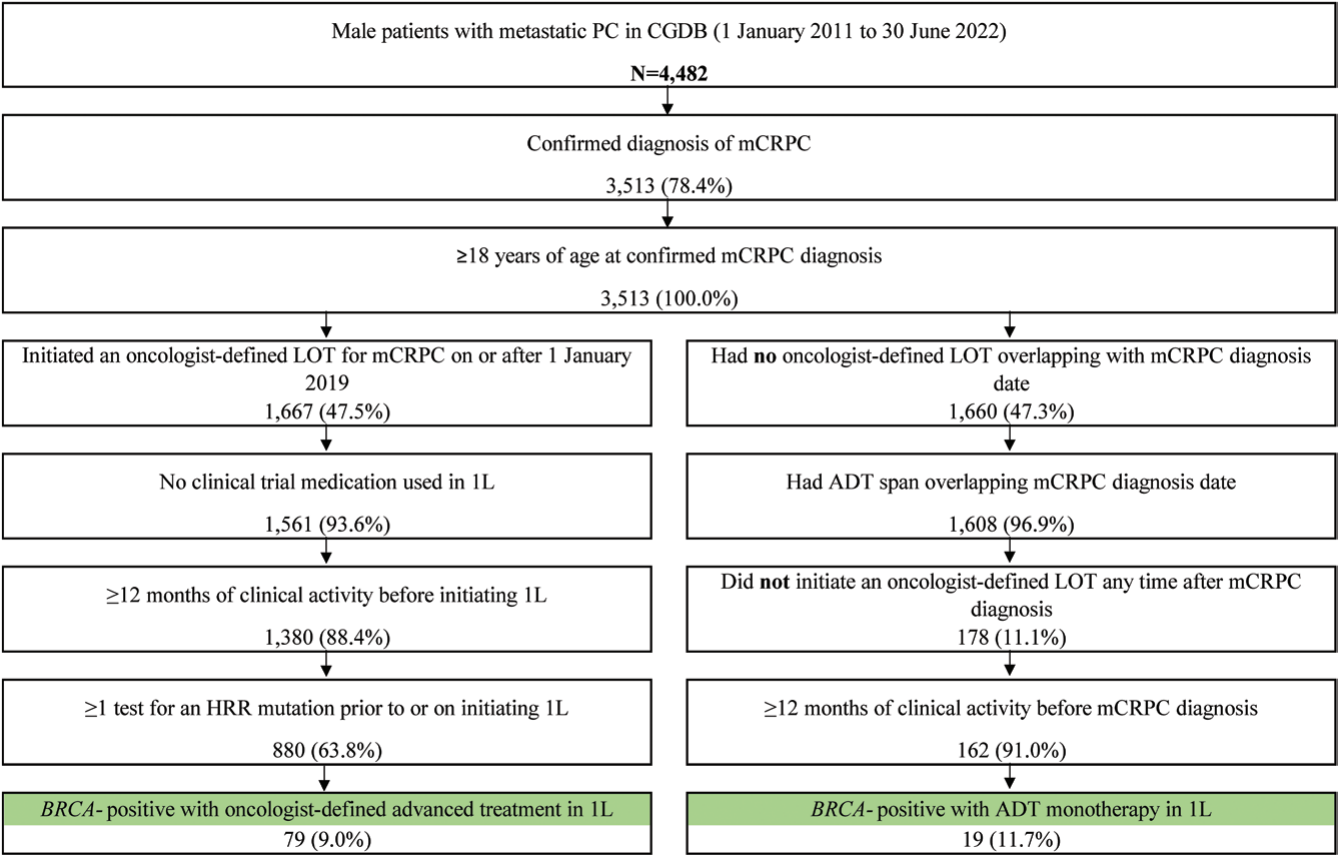
Selection of the 1L mCRPC study population. Abbreviations: 1L: first-line; ADT: androgen deprivation therapy; CGDB: Clinico-Genomic Database; HRR: homologous recombination repair; LOT: line of therapy; mCRPC: metastatic castration-resistant prostate cancer; PC: prostate cancer.

Overall, 98 *BRCA*-positive patients initiating 1L therapy for mCRPC were identified, of whom 79 (80.6%) started an advanced LOT on or after the date of mCRPC diagnosis, and 19 (19.4%) patients were using ADT monotherapy at mCRPC diagnosis (Figure 1). In the overall *BRCA*-positive treated cohort, the mean age was 72 years, 58.2% were White, 15.3% were Black, 78.6% were treated in the community setting, and 40.8% were metastatic at initial PC diagnosis (Table 1). In the ADT monotherapy subgroup, there was a greater proportion of Black patients (26.3%) and a lower proportion of patients treated in the community setting (57.9%) observed relative to the advanced LOT subgroup.

**Table 1.** Baseline demographic and clinical characteristics.

		By 1L therapy subgroup	
Demographic and clinical characteristics	Overall 1L *BRCA*-positive treated cohort	Advanced LOT	ADT monotherapy
	*N* = 98	N = 79	*N* = 19
Age, years, mean ± SD [median]	72 ± 9 [74]	73 ± 8 [74]	70 ± 10 [69]
Race, *n* (%)			
White	57 (58.2)	47 (59.5)	10 (52.6)
Black	15 (15.3)	10 (12.7)	5 (26.3)
Asian	3 (3.1)	2 (2.5)	1 (5.3)
Other	14 (14.3)	13 (16.5)	1 (5.3)
Unknown	9 (9.2)	7 (8.9)	2 (10.5)
Insurance plan type, *n* (%)			
Medicare	54 (55.1)	45 (57.0)	9 (47.4)
Commercial	27 (27.6)	23 (29.1)	4 (21.1)
Dual coverage	4 (4.1)	3 (3.8)	1 (5.3)
Medicaid	1 (1.0)	1 (1.3)	0 (0.0)
Unknown	12 (12.2)	7 (8.9)	5 (26.3)
Practice type, *n* (%)			
Community only	68 (69.4)	57 (72.2)	11 (57.9)
Academic only	21 (21.4)	13 (16.5)	8 (42.1)
Academic and community	9 (9.2)	9 (11.4)	0 (0.0)
Disease stage at initial PC diagnosis, *n* (%)			
Localized PC	58 (59.2)	45 (57.0)	13 (68.4)
mCSPC	40 (40.8)	34 (43.0)	6 (31.6)
Prior evidence of ADT use^a^, *n* (%)	97 (99.0)	78 (98.7)	19 (100.0)
Time from mCRPC diagnosis to initiation of advanced LOT^b^, days, mean ± SD [median]	-	225 ± 446 [42]	-
Year of mCRPC diagnosis, *n* (%)			
2017 or prior	12 (12.2)	6 (7.6)	6 (31.6)
2018	10 (10.2)	7 (8.9)	3 (15.8)
2019	25 (25.5)	21 (26.6)	4 (21.1)
2020	21 (21.4)	19 (24.1)	2 (10.5)
2021	22 (22.4)	19 (24.1)	3 (15.8)
2022	8 (8.2)	7 (8.9)	1 (5.3)
Type of mutation^c^, *n* (%)			
Germline	21 (21.4)	19 (24.1)	2 (10.5)
Somatic	18 (18.4)	14 (17.7)	4 (21.1)
Unknown	44 (44.9)	36 (45.6)	8 (42.1)
Missing	40 (40.8)	32 (40.5)	8 (42.1)
Most recent ECOG performance score, *n* (%)			
0	29 (29.6)	27 (34.2)	2 (10.5)
1	32 (32.7)	29 (36.7)	3 (15.8)
2	4 (4.1)	4 (5.1)	0 (0.0)
Not available	33 (33.7)	19 (24.1)	14 (73.7)
Gleason score at initial PC diagnosis, *n* (%)			
≤6	5 (5.1)	3 (3.8)	2 (10.5)
7	11 (11.2)	7 (8.9)	4 (21.1)
8	23 (23.5)	16 (20.3)	7 (36.8)
9	27 (27.6)	24 (30.4)	3 (15.8)
10	13 (13.3)	13 (16.5)	0 (0.0)
Not available	19 (19.4)	16 (20.3)	3 (15.8)
Pre-index treatment use^d^, *n* (%)			
First-generation anti-androgens	57 (58.2)	44 (55.7)	13 (68.4)
Abiraterone acetate	50 (51.0)	47 (59.5)	3 (15.8)
Localized PC therapy	49 (50.0)	37 (46.8)	12 (63.2)
Next-generation androgen receptor inhibitors	49 (50.0)	46 (58.2)	3 (15.8)
Bone antiresorptive therapy	27 (27.6)	25 (31.6)	2 (10.5)
Chemotherapy	14 (14.3)	14 (17.7)	0 (0.0)

^a^Prior evidence of ADT use was defined as any ADT at any time prior to (and excluding) the index date as observed in the “enhanced_metpc_adt” data table. Details on the specific ADT used are not available.

^b^Time from mCRPC diagnosis to initiation of 1L therapy was only reported among patients with an advanced LOT.

^c^Mutation types were not mutually exclusive as patients could have multiple mutations tests or ≥2 different mutations.

^d^Treatments received were reported any time in the period of clinical activity prior to the index date.

Abbreviations: 1L, first-line; 2L, second-line; 3L, third-line; ADT, androgen deprivation therapy; ECOG, Eastern Cooperative Oncology Group; HRR, Homologous Recombination Repair; LOT, line of therapy; mCSPC, metastatic castrate-sensitive prostate cancer; mCRPC, metastatic castrate-resistant prostate cancer; PC, prostate cancer.

### 1L mCRPC treatment

In the overall *BRCA*-positive treated cohort (*n* = 98), most patients (85.7%) were treated with monotherapy, while 14.3% of patients received therapy with more than one agent (Table 2). Other than ADT monotherapy, the most used treatments for mCRPC in 1L were enzalutamide (14.3%), olaparib (13.3%), abiraterone acetate (11.2%), and docetaxel (10.2%).

**Table 2.** 1L treatment characteristics.

Top 5 lines of therapy used, *n* (%)	Overall 1L *BRCA*-positive treated cohort *N* = 98
ADT monotherapy	19 (19.4)
Enzalutamide	14 (14.3)
Olaparib	13 (13.3)
Abiraterone acetate	11 (11.2)
Docetaxel	10 (10.2)
Therapy with more than one agent, *n* (%)	14 (14.3)

Abbreviations: 1L, first-line; ADT, androgen deprivation therapy.

### Treatment sequences

**Figure 2.**
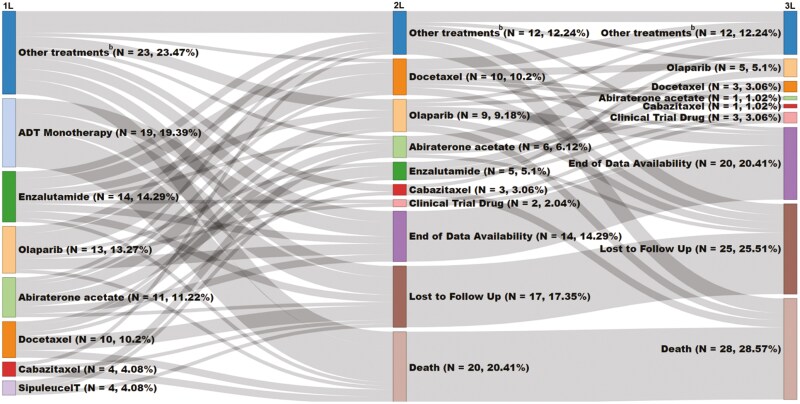
Treatment sequences for *BRCA*-positive patients with mCRPC initiating 1L therapy^a,b^. a. Mutation testing patterns were assessed during the time period any time prior to and including 1L start date among patients with an advanced lines of therapy. Advanced lines of therapy were renumbered such that 1L was defined as the first line of therapy started on or after mCRPC diagnosis with each subsequent treatment leading to an increase in the line of therapy (2L, 3L, etc.). If a patient had ≥2 HRR tests conducted within 4 weeks, these tests were grouped together when assessing test result/HRR status. Mutation testing patterns were assessed at any time during the period of continuous clinical activity among ADT monotherapy patients. b. “Other treatments” were defined as those that were used by less than 4 patients in 1L, or not used in 1L, and include: Abiraterone acetate/Olaparib, Apalutamide, Radium-223, Cabazitaxel/Carboplatin, Carboplatin, Doxorubicin, Rucaparib, Abiraterone acetate/Sipuleucel-T, Apalutamide/Sipuleucel-T, Atezolizumab/Olaparib, Carboplatin/Docetaxel, Carboplatin/Etoposide, Cyclophosphamide, Darolutamide/Olaparib, Enzalutamide/Radium-223, Enzalutamide/Sipuleucel-T, Fluouraciul/Leucovorin, Ipilimumab/Nivolumab, Lurbinectdein, Mitoxantrone, Niraparib, Olaparib/Pembrolizumab, Olaparib/Radium-223, Paclitaxel, Pembrolizumab/Rucaparib, Topotecan, Vinorelbine. Abbreviations: 1L, first-line; 2L, second-line; 3L, third-line; ADT, androgen deprivation therapy; HRR, homologous recombination repair; mCRPC, metastatic castration-resistant prostate cancer.

**Figure 3.**
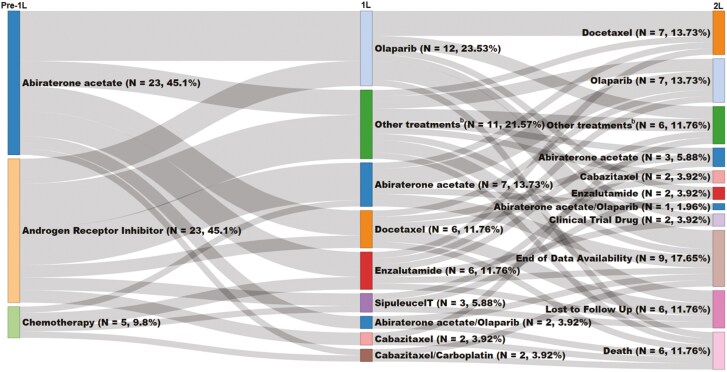
Treatment sequences among *BRCA*-positive patients by immediate prior treatment before mCRPC^a,b^. a. Treatment sequences were reported among patients with use of abiraterone acetate, androgen receptor inhibitor (ie, apalutamide, darolutamide, enzalutamide), or chemotherapy immediately prior to the 1L start date. b. “Other treatments” were defined as those that were used by less than 2 patients in 1L, or not used in 1L, and include: apalutamide/sipuleucel-T, niraparib, enzalutamide/sipuleucel-T, abiraterone acetate/olaparib/sipuleucel-T, carboplatin, fluorouracil/leucovorin/oxaliplatin, abiraterone acetate/sipuleucel-T, apalutamide, carboplatin/etoposide, pembrolizumab, enzalutamide/radium-223, paclitaxel, olaparib/radium-223, fluorouracil/leucovorin/oxaliplatin/trastuzumab, topotecan, carboplatin/docetaxel. Abbreviations: 1L, first-line; 2L, second-line; ADT, androgen deprivation therapy; HRR, homologous recombination repair; mCRPC, metastatic castration-resistant prostate cancer.

The treatment sequences for the overall *BRCA*-positive treated cohort are provided in Figure 2. Among the subgroup of patients with an advanced LOT (*n* = 79), 43.0% (*n* = 34) did not initiate a 2L of therapy, of which 35.3% (*n* = 12) were lost to follow up, and 29.4% (*n* = 10) were each censored at the end of data availability or died, and 5.9% (*n* = 2) initiated a clinical trial drug (Supplementary Table S1). For the ADT monotherapy subgroup (*n* = 19), the main reasons for censoring were death (52.6%), loss to follow up (26.3%), and end of data availability (21.1%; Supplementary Table S1). Among patients with an advanced LOT who initiated 2L (*n* = 45), the most common 1L to 2L treatment sequences were olaparib to docetaxel (11.1%), enzalutamide to docetaxel (6.7%), abiraterone acetate to enzalutamide (6.7%), and abiraterone acetate to olaparib (6.7%). The most commonly prescribed 2L therapies were docetaxel (22.2%), olaparib (20.0%), abiraterone acetate (13.3%), and enzalutamide (11.1%). In patients receiving 2L, 51.1% (*n* = 23) did not initiate 3L therapy. Among patients who used an androgen receptor inhibitor (ie, apalutamide, enzalutamide, or darolutamide) in the baseline period immediately prior to 1L mCRPC initiation (*n* = 23), most initiated abiraterone acetate in 1L mCRPC (26.1%; Figure 3). Among patients who used abiraterone acetate immediately prior to 1L mCRPC (*n* = 23), most initiated olaparib in 1L mCRPC (34.8%). Among the subgroup of patients with an advanced LOT (*n* = 79), 43 (54.4%) were treated with a PARP inhibitor at any time after mCRPC diagnosis.

### Time-to-next treatment

**Figure 4.**
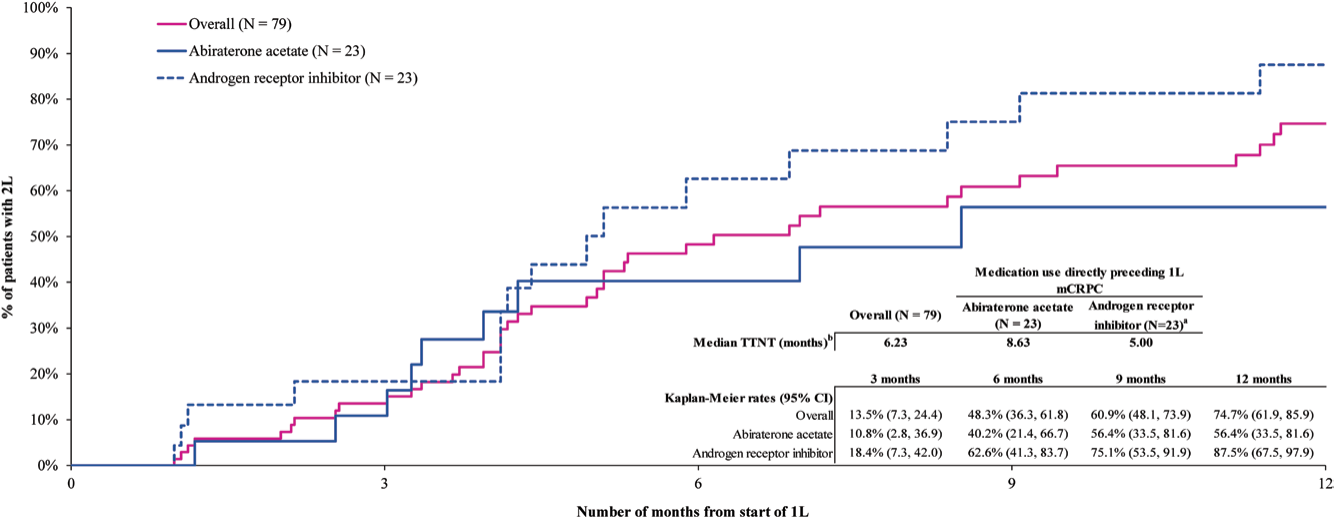
Time-to-next-treatment among *BRCA*-positive patients initiating an advanced LOT by immediate prior treatment before mCRPC^a,b,c^. a. Patients with prior use of androgen receptor inhibitors as prior treatment before mCRPC included apalutamide, enzalutamide, or darolutamide. b. Time-to-next-treatment was defined as the time from 1L start date to 2L start date (including clinical trial medications). c. Refers to the population at risk for having the event at that point in time (ie, patients who did not have the event and were not lost to follow up). Time-to-next-treatment was censored at the earliest of (1) end of clinical activity or (2) the end of data availability. Abbreviations: 1L, first-line; 2L, second-line; LOT, line of therapy; mCRPC, metastatic castration-resistant prostate cancer; TTNT, time-to-next-treatment.

Among patients with an advanced LOT in 1L (*n* = 79), the median TTNT (ie, 2L) for *BRCA*-positive patients was 6.2 months (Figure 4). Kaplan-Meier rates of 2L initiation were 13.5% at 3 months, 48.3% at 6 months, and 74.7% at 12 months. At 12 months, rates of 2L initiation were greater among patients whose used an androgen receptor inhibitor (87.5%) than patients who used abiraterone acetate (56.4%) immediately prior to 1L mCRPC.

## Discussion

This retrospective study evaluated medication use, treatment patterns, and outcomes of US patients with mCRPC with *BRCA* alterations in real-world oncology practices using the Flatiron CGDB database. Although the association between PC and *BRCA* (in particular *BRCA2*) has been known for decades,^33,34^ recent studies of *BRCA* in PC have focused on survival outcomes in patients with *BRCA* alterations compared to wild type *BRCA,* providing evidence of the high clinical burden and unmet need for treatment in these patients. A recent systematic review and meta-analyses reported a statistically significant greater risk of death using overall survival (OS) data in *BRCA2* carriers compared with non-carriers with a hazard ratio (HR) = 2.21 (95% CI: 1.64-2.99; *P* < 0.001).^20^ The present study adds to the literature by providing insights into medication use and treatment patterns in this patient population.

PARP inhibitor medications are targeted therapies developed to treat HRR deficient (eg, *BRCA*-positive) mCRPC, with olaparib and rucaparib obtaining FDA approval in 2020,^35^ and talazoparib in combination with enzalutamide^36^ and niraparib/abiraterone (dual action tablet)^37^ receiving FDA approval in June and August 2023, respectively. In the present study which enrolled patients through June 2022, olaparib was the only PARP inhibitor known to target *BRCA*-positive mCRPC observed in this population, representing the third most frequent medication used in 1L and a commonly elected agent in 2L. The remainder of patients who initiated an advanced LOT in 1L agent were treated with commonly used agents for mCRPC, which included enzalutamide, abiraterone acetate, and docetaxel. In this respect, results were similar to 2 recent real-world treatment pattern studies with abiraterone acetate, enzalutamide, and docetaxel as the most common 1L treatments in patients with mCRPC who had not undergone *BRCA* testing,^10,38^ as well as in a nationwide cohort of veterans with mCRPC, 20% of whom had undergone HRR testing.^39^ The similarity between use of more conventional (ie, non-PARP inhibitors) treatments in this study compared with recent real-world evidence studies is not surprising considering the data cutoff in the present study occurred prior to the FDA approval of talazoparib and niraparib and within a year of the FDA approval for olaparib, the only PARP inhibitor among top treatments and subsequent sequences to 2L.

In the current study, most patients (81%) were treated with an advanced LOT in 1L, while the remainder were treated with ADT monotherapy. This observation was consistent with a recent analysis of Medicare recipients with mCRPC where it was reported that 22% of patients did not receive any life prolonging therapy after mCRPC diagnosis.^10^ Prior studies have suggested that advanced age, higher comorbidity burden, contraindications with other medications, and poorer prognosis (eg, higher Eastern Cooperative Oncology Group performance status) may explain this proportion of patients who do not use advanced PC-related treatment.^10,40^ Furthermore, many patients were observed to progress to subsequent treatments in 2L and beyond, suggesting an unmet treatment need in this population. Indeed, several real-world studies in the US have assessed the large proportion of patients with mCRPC who have cycled through multiple LOTs before the availability of PARP inhibitors, which have ranged between 42% and 55% and its association with poorer clinical outcomes in this population.^10,12,40^ In the current study, TTNT was short (6.2 months) and nearly a third of patients treated with an advanced LOT who did not progress to 2L were censored due to death, suggesting elevated rates of disease progression that have been observed in other settings for patients with mCRPC, with or without *BRCA* mutations.^41^

The treatment landscape for 1L treatment in mCRPC is evolving, with niraparib plus abiraterone acetate (dual action tablet) with prednisone as the latest PARP inhibitor to be FDA-approved in August 2023, based on results from MAGNITUDE, the pivotal phase III randomized, double-blind study (NCT03748641) which showed that niraparib combined with abiraterone acetate plus prednisone significantly lengthened radiographic progression-free survival (rPFS) in patients with *BRCA*-positive mCRPC compared with standard-of-care abiraterone acetate plus prednisone by nearly 9 months.^42^ Similarly, olaparib in combination with abiraterone acetate plus prednisone has also received FDA approval in May 2023 for the treatment of *BRCA*-positive mCRPC based on results from phase III PROpel trial (NCT03732820), which demonstrated significant improvement in rPFS for the olaparib with abiraterone acetate arm (median rPFS not reached) relative to the placebo with abiraterone acetate arm (median rPFS 8 months).^43^ Furthermore, talazoparib in combination with enzalutamide was approved in June 2023 for the treatment of HRR-positive mCRPC based on results from the TALAPRO-2 trial (NCT03395197), demonstrating significantly longer rPFS for the talazoparib with enzalutamide arm (median rPFS not reached) relative to the placebo with enzalutamide arm (median rPFS 21.9 months).^44^ With the multiple ongoing studies of PARP inhibitor monotherapy and combination therapies, potentially opening up new treatment avenues for a patient population with a poor prognosis and limited treatment options, stringent inclusion criteria may limit the generalizability of these results to real-world patient populations.^24,45^ It is not known to what extent prior treatment exposure and sequencing impact the clinical effectiveness of new PARP inhibitor combinations, particularly in cases where there has been prior exposure to a component of the PARP inhibitor combination. As these new therapies become available, future studies building off the present one should assess how real-world treatment patterns evolve and how treatment sequences impact long-term outcomes.

### Limitations

This study is subject to limitations. First, the CGDB may contain inaccuracies or omissions (eg, diagnosis dates, treatment start dates, etc.), although these are expected to be random and impact all patients equally. Second, diagnoses or medical services obtained outside the Flatiron Health network were not captured. This would include medications received from the urology setting prior to being treated by medical oncologists in the Flatiron network. Third, patients selected from the CGDB may not be representative of the entire population of patients with mCRPC in the US, which may limit the generalizability of the study. Fourth, HRR alteration testing is subject to errors during blood/tissue sampling and laboratory procedures, which may potentially affect the selection of the *BRCA*-positive population.

## Conclusion

In this real-world study, ADT monotherapy, enzalutamide, and olaparib were most commonly used medications in patients with *BRCA*-positive mCRPC initiating treatment. Most patients who received ADT monotherapy died, while most patients receiving advanced 1L treatment progressed to 2L, notably with chemotherapy, suggesting an unmet need for patients with *BRCA*-positive mCRPC.

## Supplementary Material

oyae183_suppl_Supplementary_Table_S1

## Data Availability

Data that support the findings of this study were used under license from Flatiron Health, Inc. and Flatiron-Foundation Medicine. Restrictions apply to the availability of these data, which are not publicly available and cannot be shared. The data are available by request made directly to the data vendor, subject to the data vendor’s requirements for data access.

## Data transparency

The authors declare that the data supporting the findings of this study are available within the article and its supplementary information files.
