# Supplementary material for: Treatment patterns for patients with BRCA1/2-positive metastatic castration-resistant prostate cancer
Source: Oncologist. 2024 Jul 31;30(1):oyae183. doi: 10.1093/oncolo/oyae183 (PMC11783294; doi:10.1093/oncolo/oyae183)
Supplement: oyae183_suppl_Supplementary_Table_S1 [file oyae183_suppl_supplementary_table_s1.docx]

**Supplemental Table 1. Most common 1L→2L and 2L→3L treatment sequences and reasons for censoring**

| ***BRCA*-positive with an advanced LOT in 1L**  **N=79** | | | | | | |
| --- | --- | --- | --- | --- | --- | --- |
| **Patients progressing to 2L** | 45 | (57.0) | **Patients progressing to 3L** | 22 | | (48.9) |
| **Most common 1L → 2L treatment sequences, n (%)** |  |  | **Most common 2L → 3L treatment sequences, n (%)** |  | |  |
| Olaparib → Docetaxel | 5 | (11.1) | Abiraterone acetate → Radium-223 | 2 | | (9.1) |
| Abiraterone acetate → Enzalutamide | 3 | (6.7) | Docetaxel → Olaparib | 2 | | (9.1) |
| Abiraterone acetate → Olaparib | 3 | (6.7) | Enzalutamide → Docetaxel | 2 | | (9.1) |
| Enzalutamide → Docetaxel | 3 | (6.7) | Enzalutamide → Olaparib | 2 | | (9.1) |
| Apalutamide → Olaparib | 2 | (4.4) |  |  | |  |
| Docetaxel → Abiraterone acetate | 2 | (4.4) |  |  | |  |
| Enzalutamide → Olaparib | 2 | (4.4) |  |  | |  |
| Sipuleucel-T → Abiraterone acetate | 2 | (4.4) |  |  | |  |
| **Patients censored before 2L, n (%)** | 34 | (43.0) | **Patients censored before 3L, n (%)** | 23 | | (51.1) |
| **Reasons for censoring, n (%)** |  |  | **Reasons for censoring, n (%)** |  | |  |
| Clinical trial drug | 2 | (5.9) | Clinical trial drug | 1 | | (4.3) |
| End of data availability | 10 | (29.4) | End of data availability | 6 | | (26.1) |
| Loss to follow-up | 12 | (35.3) | Loss to follow-up | 8 | | (34.8) |
| Death | 10 | (29.4) | Death | 8 | | (34.8) |
| ***BRCA-*positive with ADT monotherapy in 1L**  **N=19** | | | | | | |
| **Patients censored at 2L, n (%)** | 19 | (100.0) |  |  |  | |
| **Reasons for censoring, n (%)** |  |  |  |  |  | |
| Death | 10 | (52.6) |  |  |  | |
| Loss to follow-up | 5 | (26.3) |  |  |  | |
| End of data availability | 4 | (21.1) |  |  |  | |

Abbreviations: 1L: first-line; 2L: second-line; 3L: third-line; ADT: androgen deprivation therapy; LOT: line of therapy.
